# Ethnicity evaluation of ferric pyrophosphate citrate among Asian and Non-Asian populations: a population pharmacokinetics analysis

**DOI:** 10.1007/s00228-022-03328-9

**Published:** 2022-06-17

**Authors:** Lingxiao Zhang, Liangying Gan, Kexin Li, Panpan Xie, Yan Tan, Gang Wei, Xiaojuan Yuan, Raymond Pratt, Yongchun Zhou, Ai-Min Hui, Yi Fang, Li Zuo, Qingshan Zheng

**Affiliations:** 1 Center for Drug Clinical Research, Shanghai University of Traditional Chinese Medicine, Shanghai, China; 2 Department of Nephrology, Peking University People’s Hospital, Beijing, China; 3 Clinical trial center, Beijing hospital, National center of gerontology, Institute of geriatric medicine, Chinese academy of medical sciences, Assessment of Clinical Drugs Risk and Individual Application Key Laboratory, Beijing, China; 4 Global R&D Center, Shanghai Fosun Pharmaceutical Development, Co, Ltd, Shanghai, China; 5 Jiangsu Wanbang Biopharmaceuticals Co., Ltd, Xuzhou, China; 6 Rockwell Medical Inc., Wixom, MI USA; 7 Department of Pharmacy, Peking University People’s Hospital, Beijing, China

**Keywords:** Ethnicity, Modeling and simulation, CKD-5HD, Population pharmacokinetics

## Abstract

**Purpose:**

To evaluate the potential ethnic differences of ferric pyrophosphate citrate (FPC, Triferic) in healthy subjects and patients with hemodialysis-dependent stage 5 chronic kidney disease (CKD-5HD) and identify covariates that may influence pharmacokinetics (PK) of FPC.

**Methods:**

Data were collected from 2 Asian and 4 non-Asian clinical studies involving healthy subjects and CKD-5HD patients. Three population PK models were developed: M1 for intravenous (IV) administration of FPC in healthy subjects; M2 for dialysate administration of FPC in CKD-5HD patients; M3 for pre-dialyzer administration of FPC in CKD-5HD patients. All the models were fitted to concentration versus time data of FPC using the nonlinear mixed effect approach with the NONMEM^®^ program. All statistical analyses were performed using SAS version 9.4.

**Results:**

In total, 26 Asians and 65 non-Asians were included in the final model analysis database. Forty healthy subjects were administered FPC via intravenous (IV) route and 51 patients with CKD-5HD via dialysate (*N* = 50) and pre-dialyzer blood circuit administration (*N* = 51). The PK parameters of FPC IV were similar. The population PK model showed good parameter precision and reliability as shown by model evaluation, and no relevant influence of ethnicity on PK parameters was observed. In healthy subjects, the maximum observed plasma concentration (C_max_) and area under the plasma concentration–time curve (AUC) decreased with increase in lean body mass (LBM) and the average serum total iron at 6 h before the baseline period (Fe_av_), whereas, in both patient populations, C_max_ and AUC decreased with increase in LBM and decrease in Fe_baseline_. Other factors such as gender, age, Fe_av_, and ethnicity had no influence on PK exposures in patients. The influence of LBM on PK exposures in patients was smaller than that in healthy subjects (ratio of AUC_0-24_ for the 5th [68 kg] and 95th [45 kg] patient’s LBM was almost 1). The influence of Fe_av_ and LBM on PK exposures was < 50%.

**Conclusion:**

The population pharmacokinetics model successfully described the PK parameters of FPC in healthy subjects and CKD-5HD patients and were comparable between Asian and non-Asian populations.

**Supplementary information:**

The online version contains supplementary material available at 10.1007/s00228-022-03328-9.

## Introduction

Iron deficiency remains a major cause of anemia in patients with hemodialysis-dependent stage 5 chronic kidney disease (CKD-5HD). These patients have enhanced iron requirements because of the use of erythropoiesis-stimulating agents (ESAs), and chronic blood loss associated with dialysis [1–3]. Therefore, intravenous (IV) iron supplementation is often provided to this patient population to increase transferrin saturation (TSAT) and serum ferritin values [4]. Most iron supplements are bound to a carbohydrate moiety and are effective in improving hemoglobin (Hgb) levels and reducing the required dose of ESAs [5, 6]. More often, these drugs are widely used for iron replacement in patients undergoing hemodialysis (HD). The European Best Practice Guidelines for the management of anemia in patients with chronic kidney disease (CKD) [7] and the National Kidney Foundation (NKF)-Kidney Disease Outcomes Quality Initiative (K/DOQI) Clinical Practice Guidelines for Anemia of CKD [8] have recommended IV iron administration in patients with CKD for achieving the target hemoglobin (Hgb), improving survival and quality of life, and reducing hospitalizations. However, all the available IV iron supplements are iron-carbohydrate complexes which are likely to promote cytotoxicity, increase risks of inflammation, exacerbate oxidative stress and endothelial dysfunction, and lead to the progression of CKD and cardiovascular disease [9–11].

Ferric pyrophosphate citrate (FPC, Triferic) is a novel carbohydrate-free, water-soluble, iron replacement agent in which citrate and pyrophosphate compounds are tightly bound to iron, reducing risk for free iron release into the blood stream [12, 13]. FPC, which was developed by Rockwell Medical, was approved as a maintenance iron supplement in the USA on January 23, 2015, to be administered via dialysate in adult patients with CKD receiving HD [14]. The major difference of FPC over other IV iron supplements is that iron is delivered via a dialysate and not injected [12]. The specific routes of administration of FPC in patients with CKD-5HD are via dialysate and via the pre-dialyzer blood circuit administration [15]. Slow infusion of soluble FPC by dialysate was safe and effective alternative to IV administration in patients undergoing hemodialysis (HD). Dialysate containing the desired concentrations of FPC were generated by adding FPC to the bicarbonate concentrate [16]. Unlike other iron supplements, FPC allows for optimal iron utilization during erythropoiesis and avoids iron sequestration within reticuloendothelial (RE) macrophages [16, 17].As FPC is highly soluble in aqueous solutions, it can also be administered intravenously. Intravenous administration allows patients receiving hemodialysis with solid bicarbonate cartridges to receive FPC iron concurrently with HD [12].

The multicenter randomized, placebo-controlled phase III clinical studies (PRIME, CRUISE 1 and 2) for FPC have found dialysate administration to maintain Hgb level and iron balance in patients receiving chronic HD [18, 19]. Adverse events were similar in both the dialysate FPC-treated and placebo groups [18, 19]. The pharmacokinetic (PK) profile of FPC has been investigated in healthy subjects and pediatric subjects with HD in previous research, [12, 20] which was mostly conducted in Caucasian and Asian patients.

Owing to the differences in genetic, physiological, and pathological factors between ethnic/racial groups, it is possible for PK parameters of drugs to vary with ethnicity [21, 22]. To date, no analyses have evaluated the influence of ethnicity on PK parameters of FPC. Hence, in the current analysis, we established a population PK model by combining data from studies performed in Asians, particularly Chinese and non-Asians, including African American and Caucasian populations. Population PK modeling was used to evaluate potential ethnic differences of FPC among Asian and non-Asian subjects for 3 different routes of administration: IV administration in healthy subjects, dialysate, and pre-dialyzer administration in patients with CKD-5HD.

## Materials and methods

### Description of clinical studies

A consolidated dataset for analysis was generated by combining PK data following FPC administration via the IV route in healthy subjects, via dialysate in patients with CKD-5HD, and via the pre-dialyzer blood circuit administration in patients with CKD-5HD. The route of administration might influence the PK parameters of the drug; hence, the influence of route of administration in PK parameters was evaluated for which data was available from Asian, particularly Chinese, and non-Asian including African American and Caucasian populations. Data were collected from 6 clinical studies: clinical studies conducted in Chinese healthy subjects (CHN-FPC-14, CTR20181113) and patients with CKD-5HD (CHN-FPC-21, CTR20181119) and clinical studies conducted in non-Asian healthy subjects (USA-FPC-12, NCT02636049 and USA-FPC-18, NCT02767128) and patients with CKD-5HD (USA-FPC-16, NCT02739100 and USA-FPC-20, NCT02767128). Source data for this analysis were provided by Jiangsu Wanbang Biopharmaceuticals Co., Ltd., and Rockwell Medical Inc.

Details on dosage regimen and blood sample collection after FPC administration of the included studies is provided in Online Resource 1 and the design of our study is presented in Online Resource 2.

All included studies were conducted under the principles of the Declaration of Helsinki and the Good Clinical Practice guidelines of the International Conference on Harmonization. All participants provided written informed consent before enrollment in each study.

### Pharmacokinetics data analysis

Population PK analysis was performed using 3 datasets, namely, M1, healthy individuals; M2, CKD-5HD patients treated with FPC dialysate; and M3, CKD-5HD patients treated with pre-dialyzer FPC (administration of FPC through pre-dialysate blood circuit for 3 h). Three separate models were established to accurately quantify the effects of covariates such as ethnic differences and to achieve the research purposes.

There are differences in PK parameter under different dosing regimens; hence, in this analysis, 3 different models were established to accurately determine the influence of ethnicity on PK parameters. All statistical analyses were performed using SAS (version 9.4, Cary, North Carolina, USA).

### Base model development

All model parameters were estimated using the first-order conditional estimation with interaction (FOCEI) method using nonlinear mixed effects modelling software (NONMEM, version 7.3, ICON Development Solutions, Ellicott City, MD, USA) [23]. The PK of FPC was determined by one-compartment base model parameterized in terms of apparent clearance (CL for FPC administration via IV route and CL/F for FPC administration via dialysate and pre-dialyzer) and apparent volume of distribution (V_d_ for IV route and V_d_/F for dialysate and pre-dialyzer administration) (Supplementary Fig. 1). The Akaike information criterion (AIC) with a threshold of 2 (model in which the difference in AIC relative to AIC_min_ was < 2 will be considered) was used to choose the base model [24].

### Building random effects model

Random effects were accounted by including inter-individual variability for PK parameters and residual error for iron concentrations. Inter-individual variability in PK parameters was characterized using an exponential model according to the following relationship:

1 $${P}_{i} = {P}_{TV}\cdot exp \;(\eta i)$$

where *P*_*i*_ is the estimated parameter for individual *i*, *P*_*TV*_ is the typical population value of the parameter, and *η*_*i*_ is the inter-individual random effect variable for individual *i* and is assumed to be normally distributed with a mean of 0 and variance of ω^2^.

The difference between the model-predicted total iron plasma concentrations and observed concentrations was modeled using a residual error model. Residual error models accounted for error in the bioanalytical assay, sample collection, and model misspecification. Residual variability was evaluated using 3 different residual error (ε) models: additive Eq. 2), proportional Eq. 3), and combined additive and proportional (Eq. 4) error models.

2 $${Y}_{obs,ij} = {Y}_{pred,ij}+ {\upvarepsilon }_{ij,2}$$

3 $${Y}_{obs,ij} = {Y}_{pred,ij} \times (1 + {\upvarepsilon }_{ij,1})$$

4 $${Y}_{obs,ij} = {Y}_{pred,ij} \times (1 + {\upvarepsilon }_{ij,1}) + {\upvarepsilon }_{ij,2}$$

where *Y*_*obs,ij*_ and *Y*_*pred,ij*_ are the observed and predicted total iron concentrations in the ith individual at time j, *ε*_*ij,1*_ is a random variable for the proportional error model with a mean of 0 and variance of *σ*_*1*_^*2*^, *ε*_*ij,2*_ is a random variable for the additive error model with a mean of 0 and variance of *σ*_*2*_^*2*^.

### Covariate model development

Covariate evaluation included gender, age, average serum total iron at 6 h before the baseline period [Fe_av_], serum total iron at 0 h before administration [Fe_baseline_], lean body mass [LBM], red blood cell [RBC] count, hemoglobin [Hgb], serum creatinine clearance rate [CrCL], aspartate aminotransferase [AST], alanine aminotransferase [ALT], albumin [ALB], total bilirubin [TB], total cholesterol [TC], C-reactive protein [CRP], and ethnicity. As the Fe_basleine_ of FPC is not 0, it might affect the PK parameters; hence, the influence of Fe_av_ in FPC IV administration and Fe_baseline_ in administration via dialysate and pre-dialyzer administration dataset were evaluated. First, a graphical approach was used to examine the correlation between covariates. If the covariates showed significant correlation (correlation coefficient > 0.8), only the clinically meaningful covariates were included into the final covariate model to avoid collinearity (Online Resource 3). After the base model was developed, correlation scatter plots were generated using Empirical Bayes (EB) estimate of individual PK parameter and covariates. The final covariates evaluated were determined based on the trends of correlation scatter plots and the physiological significance of the covariates [25].

The influence of covariates on the CL or CL/F and V_d_ or V_d_/F were tested. Eqs. 5 and 6 were applied to test continuous covariates using fractional and power models, and categorical covariates were modeled as shown in Eq. 7.

5 $${P}_{i} = {P}_{TV} \times [1 + {\theta }_{k} \times ({COV}_{k} - {COV}_{median})]$$

6 $${P}_{i} = {P}_{TV} \times {\left(\frac{{COV}_{k}}{{COV}_{median}}\right)}^{\uptheta }$$

where *P*_*i*_ is the PK parameter estimate in the ith individual and *COV*_*k*_ is the kth continuous covariate centered or normalized by the median covariate value (COV_median)_, *P*_*TV*_ is the typical population value of a parameter, *θ*_*k*_ represents the fractional change or exponent of the power model associated with the kth covariate.

7 $${P}_{i}= \left\{\begin{array}{c}{P}_{TV,} \;if \;Category=1\\ \\ { P}_{TV}\times \left(1+ \theta \right), \;if\; Category=2\end{array}\right.$$

where *P*_*i*_ is the parameter estimate in the ith individual, *θ* is the categorical covariate coefficient, and *P*_*TV*_ is the typical population value of a parameter for one category.

### Forward inclusion to establish full covariate model

Covariates were introduced into the final base model in a stepwise manner. A likelihood ratio test was used to evaluate the statistical significance of addition of each covariate to the model. Covariates with significant improvements (*P* < 0.05) in the objective function value (OFV, a decrease in OFV > 3.84) were retained in the model. This procedure was repeated, adding each covariate relationship individually, until all significant covariates were identified to establish the full covariate model.

#### Backward elimination to establish the final covariate model

Following forward addition, covariates were then removed one-by-one from the full covariate model to determine if they should be retained. A covariate was retained in the model if its removal was associated with a significant increase in the OFV (OFV increase of > 6.63, *P* < 0.01). The remaining non-significant covariates were removed from the model. The final model was obtained after completion of the backward elimination process. No correlation > 0.95 between individual random effect parameters (covariates) should be present in the final model.

### Model evaluation

Various goodness of fit (GOF) diagnostics were used to evaluate the quality of the model fit to the data. Diagnostics included GOF plots of observed concentrations versus population predicted and individual predicted concentrations, CWRES versus population predicted concentrations, and weighted residuals versus time. To be considered reliable, relative standard error of model parameter estimates should be < 50%. Bootstrap runs (*n* = 1000) were performed to provide point estimates and precision estimates of PK parameters for the final model. Point estimates for each parameter were calculated as the median value across bootstrap runs. In addition, the successful rate of bootstrapping method and precision of the parameter estimates derived from bootstrapping were used to evaluate the robustness of the final model by re-sampling the original data for 1000 times to obtain 1000 new datasets and parameters for each dataset were estimated. Non-parametric bootstrap estimate for each parameter was calculated for the median and 95% percentile interval (PI) of the 1000 simulations, which is the 2.5th and 97.5th percentile of the 1000 bootstrapping estimates of the parameter. The parameter estimates from the original data were compared to the 95% CI of those estimated from bootstrapping method.

Furthermore, a visual prediction check (VPC) was used to evaluate the performance of the final pop PK model by simulating 5th, 50th, and 95th percentile of FPC concentration time data for each of 1000 simulations of the final dataset with the final pop PK model. The 95% confidence interval of 5th, 50th, and 95th percentiles from the 1000 simulations were compared to that of observed 5th, 50th, and 95th percentiles of the actual data.

Differences in PK between Asian and non-Asian populations were evaluated. The effects of covariates retained in the final population PK model on total iron exposure were also evaluated. A geometric mean ratio of 0.8 to 1.25 for PK parameters between two ethnic groups indicated no influence of ethnicity, whereas the ratio beyond this range showed ethnic difference for PK parameters [26].

## Results

### Demographics

The total population including Asians (*N* = 26) and non-Asians (*N* = 65) was 91, out of which 40 healthy subjects were administered FPC via IV route and the remaining 51 patients with CKD-5HD were given FPC via dialysate and pre-dialyzer blood circuit administration. There were 14 healthy Asians from CHN-FPC-14, 12 healthy non-Asians from USA-FPC-12, and 14 healthy non-Asians from USA-FPC-18 study, all of whom were administered FPC via IV route and included in the final model analysis database (M1). In the FPC administration via dialysate dataset, there were a total of 50 patients with CKD-5HD (one patient of USA-FPC-20 lacked PK data of dialysate administration) consisting of 12 Asians from CHN-FPC-21, 25 and 13 non-Asians from USA-FPC-20 and USA-FPC-16 study, respectively, who received FPC via dialysate (M2). And all the 51 patients with CKD-5HD consisted the pre-dialyzer blood circuit administration dataset (12 Asians from CHN-FPC-21, 26 and 13 non-Asians from USA-FPC-20 and USA-FPC-16 who received pre-dialyzer administration of FPC) (M3). The baseline characteristics of patients in all the studies are presented in Table 1, and the baseline demographics each study used for the development of M1, M2, and M3 is presented in Online Resource 4,

**Table 1:** Baseline demographic characteristics of each study

	**Asians (*N* = 26)**		**Non-Asians (65)**			
	**Healthy subjects**	**Patients with CKD-5HD**	**Healthy subjects**	**Healthy subjects**	**Patients with CKD-5HD**	**Patients with CKD-5HD**
	**CHN-FPC-14** **(*N* = 14)**	**CHN-FPC-21** **(*N* = 12)**	**USA-FPC-12** **(*N* = 12)**	**USA-FPC-18** **(*N* = 14)**	**USA-FPC-16** **(*N* = 13)**	**USA-FPC-20** **(*N* = 26)**
***Age (year)***						
Mean ± SD	30.8 ± 5.9	54.3 ± 16.4	38.4 ± 8.9	39.1 ± 11.9	49.2 ± 8.8	53.8 ± 8.2
Min, max	19, 40	25, 77	24, 54	21, 62	34, 63	36, 68
***Sex***						
Male/female	13/1	9/3	10/2	12/2	11/2	21/5
***Weight (kg)***						
Mean ± SD	68.1 ± 6.9	69.3 ± 10.2	84.4 ± 12.8	73.7 ± 12.9	97.7 ± 19.9	84.9 ± 14.4
Min, max	57.3, 76.6	56.7, 91.1	65, 104.9	53.3, 95.3	65, 129	51.3, 122.8
***RBC (*× *10***^***12***^***/L)***						
Mean ± SD	5.08 ± 0.3	3.7 ± 0.4	4.8 ± 0.4	4.7 ± 0.3	4.0 ± 0.5	3.9 ± 0.5
Min, max	4.4, 5.5	3.1, 4.5	4.0, 5.4	4.2, 5.5	3.3, 5.4	3.0, 4.8
***Hgb (g/dL)***						
Mean ± SD	15.7 ± 1.0	11.9 ± 1.2	14.5 ± 1.2	14.6 ± 0.8	12.3 ± 1.2	11.7 ± 1.5
Min, max	13.3, 18	9.9, 14.4	12.9, 16.6	13.2, 16.1	10.5, 15.2	9.2, 14.3
***PLT***						
Mean ± SD	267.9 ± 54.2	188.3 ± 50.3	220.5 ± 52.0	226.9 ± 36.3	154.8 ± 46.3	190 ± 67
Min, max	167, 366	107, 244	148, 333	152, 272	87, 252	94, 339
***CrCL (mL/min)***						
Mean ± SD	137.7 ± 20.7	6.9 ± 1.0	114.5 ± 22.8	103.7 ± 21.8	14.4 ± 6.4	12.5 ± 4.4
Min, max	98.7, 166.0	5.5, 8.9	93.1, 176.5	64.9, 162.1	6.9, 28.0	6.6, 26.5
***AST(U/L)***						
Mean ± SD	19.1 ± 4.0	14.2 ± 3.5	19.2 ± 3.1	21.1 ± 4.5	21.2 ± 12.2	18.1 ± 5.1
Min, max	13, 27	10, 23	14, 23	15, 29	11, 54	10, 33
***ALT (U/L)***						
Mean ± SD	19.9 ± 10.0	13.6 ± 7.2	21.8 ± 6.2	21.6 ± 8.1	22.9 ± 19.8	13.0 ± 6.8
Min, max	8, 44	4, 28	13, 30	10, 41	10, 84	3, 33
***ALP (U/L*)**						
Mean ± SD	84.6 ± 16.2	91.9 ± 29.46	63.3 ± 15.1	66.9 ± 19.5	121.9 ± 66.72	96.5 ± 40.7
Min, max	66, 115	50, 153	42, 87	39, 119	42, 313	34, 186
***TBL (μmol/L)***						
Mean ± SD	0.6 ± 0.2	0.4 ± 0.1	0.4 ± 0.2	0.6 ± 0.2	0.4 ± 0.2	0.3 ± 0.1
Min, max	0.4, 1.09	0.27, 0.76	0.2, 0.9	0.4, 1.2	0.1, 1.1	0.1, 0.7
***TC (mmol/L)***						
Mean ± SD	164.3 ± 21.2	155.9 ± 27.8	178.5 ± 25.4	188.1 ± 40.7	155.2 ± 34.6	151.6 ± 33.3
Min, max	123.7, 210.7	88.5, 185.2	140, 216	125, 264	114, 232	88, 210
**CRP (mg/dL)**						
Mean ± SD	-	0.4 ± 0.4	1.1 ± 0.4	1.4 ± 0.7	0.8 ± 0.8	0.8 ± 0.6
Min, max	-	0.07,1.4	1, 2.4	1, 3.1	0.03, 2.2	0.01, 2.3
***Baseline Fe***_***tot***_*** (CONC0) (ng/mL)***						
Mean ± SD	-	794.7 ± 222.9	-	-	594.6 ± 206.1	613.2 ± 246.1
Min, max	-	369.6, 1254.4	-	-	320, 920	210, 1100
***Fe***_***av***_*** (ng/mL)***						
Mean ± SD	946.4 ± 221.4	-	1170.7 ± 352.7	1425.5 ± 554.6	-	-
Min, max	583.1, 1250.2	-	490.7, 1732.9	693.3, 2828.6	-	-
***Fe***_***.cmax***_***(ng/mL)***						
Mean ± SD	1054.4 ± 217.1	-	1367.9 ± 402.2	1750.8 ± 648.1	-	-
Min, max	694.4, 1360.8	-	584, 1949	1074, 3301	-	-
***LBM (kg)***						
Mean ± SD	51.8 ± 5.0	50.5 ± 6.5	59.1 ± 8.7	54.0 ± 6.8	62.3 ± 8.5	57.1 ± 5.6
Min, max	37.9, 56.31	37.8, 61.1	41.7, 70.3	37.6, 64.2	48.7, 76.7	43.8, 67.7

*AL*, alanine aminotransferase, *AST* aspartate aminotransferase, *CrCL* serum creatinine clearance, *CRP* C-reactive protein, *Fe*_*.av*_ average serum total iron at 6 h before the baseline period, *Fe*_*baselin*_, serum total iron at 0 h before administration, *Hgb*, hemoglobin, *LBM* lean body mass, *RBC* red blood cell, *PLT* platelet, *SD* standard deviation, *TB* total bilirubin, *TC* total cholesterol

### Population pharmacokinetic model

**Fig. 1:**
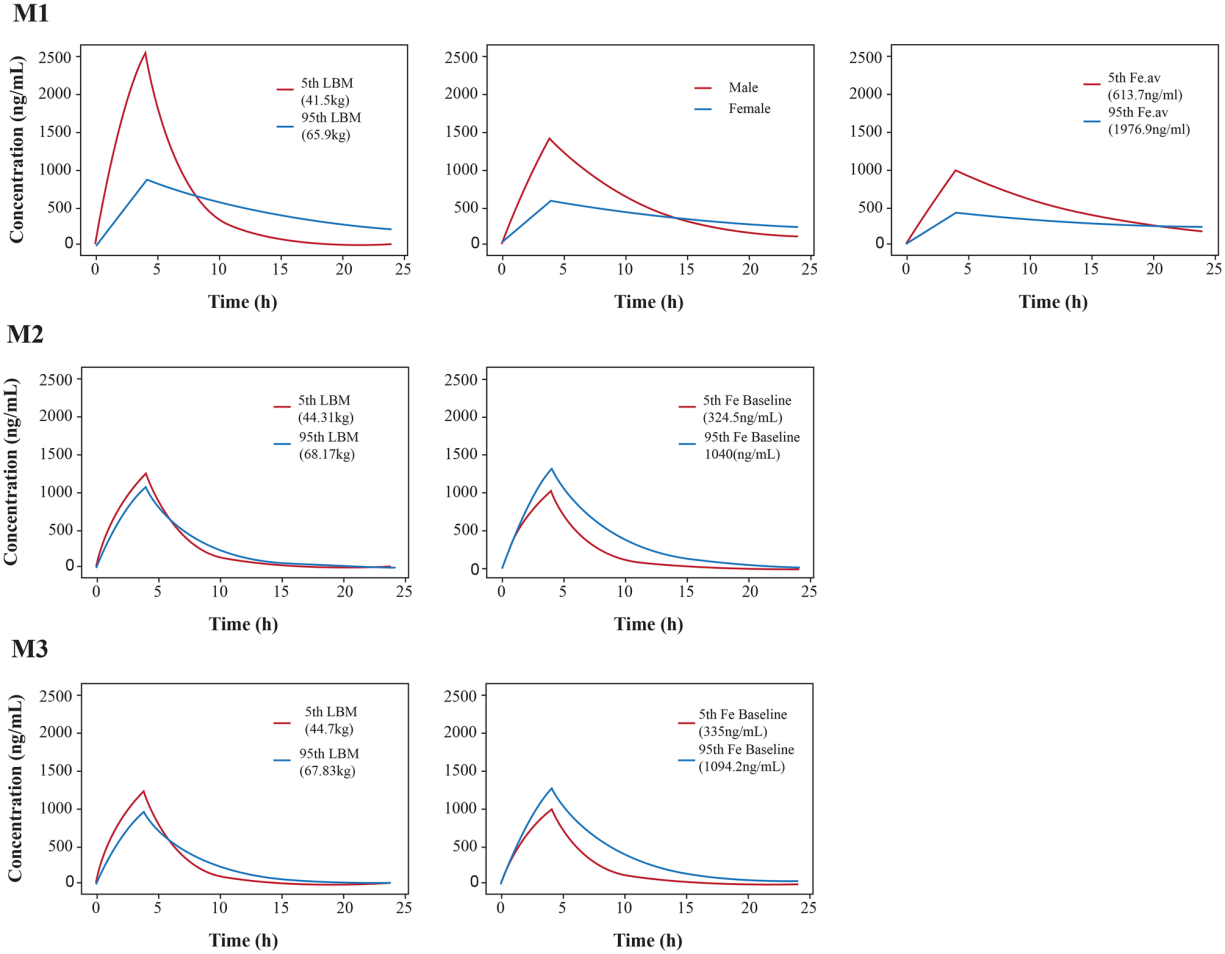
Simulated drug concentration–time curve **M1** after the administration of IV FPC 6.5 mg for 4 h in healthy subjects, **M2** after the administration of FPC 6.5 mg for 4 h via dialysate in patients with CKD-5HD, and **M3** after the pre-dialyzer administration of FPC 6.5 mg for 3 h in patients with CKD-5HD. CKD-5HD, hemodialysis-dependent stage 5 chronic kidney disease; IV, intravenous; LBM, lean body mass

**Fig. 2:**
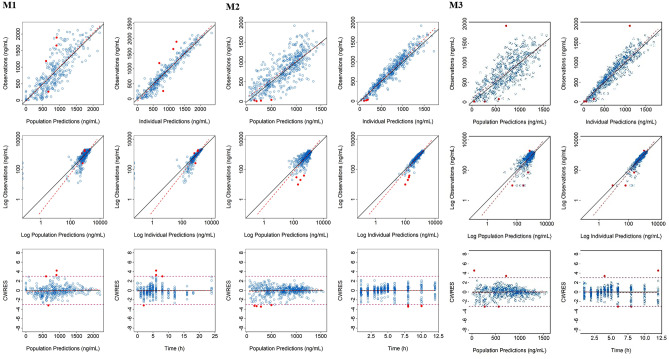
Goodness of fit of FPC in (M1) IV model of healthy subjects, (M2) dialysate model of patients with CKD-5HD, and (M3) pre-dialyzer model of patients with CKD-5HD. Blue circles represent observed or model-predicted data points; black lines represent the line of unity or horizontal line with y = 0; and red lines represent the regression lines. CWRES, conditional weighted residuals; CKD-5HD, hemodialysis-dependent stage 5 chronic kidney disease; IV, intravenous

**Fig. 3:**
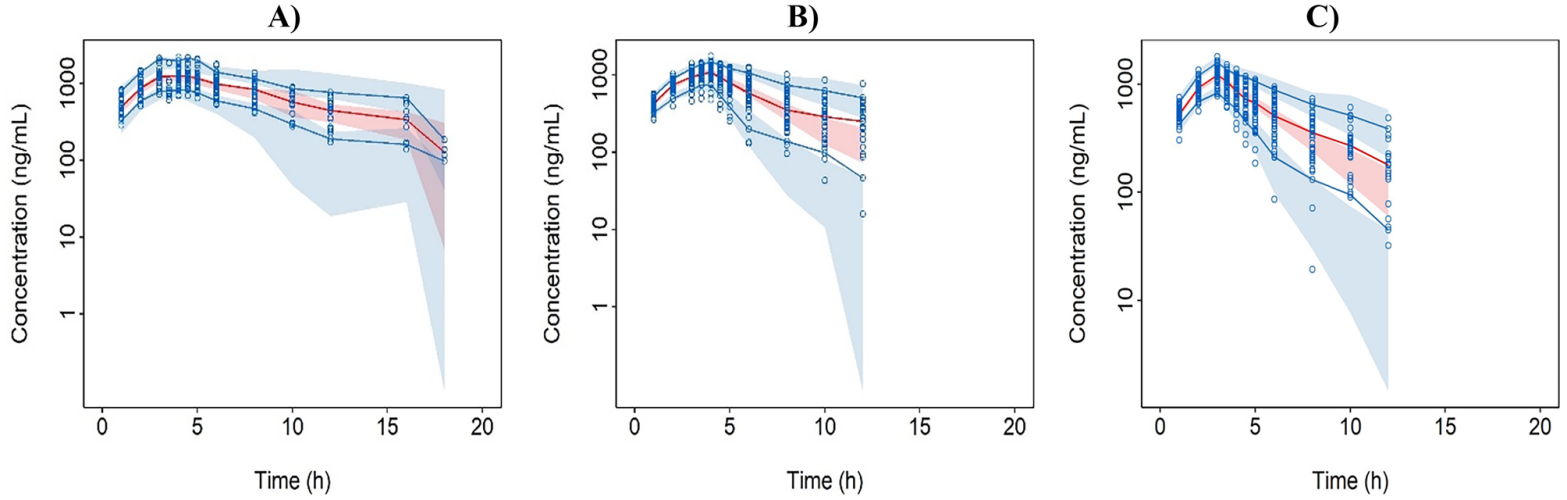
Visual predictive check of FPC in **A** IV model of healthy subjects, **B** dialysate model of patients with CKD-5HD, and **C** pre-dialyzer model of patients with CKD-5HD. Blue circles represent observed values; blue solid lines and red solid line represent 5th, 95th, and 50th percentiles of the observed data; blue and red shaded areas represent the model-predicted 95% confidence interval for the 5th, 95th, and 50th percentiles. CKD-5HD, hemodialysis-dependent stage 5 chronic kidney disease; IV, intravenous

A one-compartmental model adequately described the PK of FPC in Asians and non-Asians. The residual error in M1 was described by an additive error model, and in M2 and M3 by a combined additive and proportional error model. The inter-individual variability (RSE %) in CL and V_d_ in M1 were 42.8% (23%) and 33.8% (16.7%), respectively, while the additive residual error (RSE%) was 174 ng/ml (8.9%) in the final model. Similarly, inter-individual variability (RSE%) in CL/F and V_d_/F were 41.5% (11.9%) and 18.1% (21.0%) in M2, and 36.6% (8.9%) and 21.0% (23.2%) in M3, respectively, while the proportional residual errors (RSE%) in M2 and M3 were 23.6% (8.9%) and 27.1% (7.8%), respectively, in the final model. The inter-individual variability of both base and final models in patients was lower than that in healthy subjects. About 2–5% inter-individual variability of the base models was explained by the covariates. The residual errors in M2 and M3 were mostly described by the proportional error resulting in low additive errors in M2 and M3, while it was high in M1 which was described only by the additive error. The CL and V_d_ in FPC IV administration in healthy subjects and CL/F and V_d_/F in FPC via dialysate and pre-dialyzer in patients with CKD-5HD were similar. When FPC IV was administered in healthy subjects, the CL and V_d_ were 0.477 L/h and 3.62 L, respectively (Table 2). In the administration of FPC via dialysate in patients with CKD-5HD dataset, the CL/F was 0.982 L/h and V_d_/F was 3.32 L. Similarly, when FPC pre-dialyzer was administered in patients with CKD-5HD, the observed CL/F was 1.02 L/h and V_d_/F was 3.57 L. When FPC IV was administered in healthy subjects, except for the relative standard error (RSE)% of covariate sex on V_d_ which was 30.1%, the RSE% of other parameters was < 30%. With the increase in LBM and Fe_av_, V_d_ also increased which was 2.8 times higher in women than in men. In administration via dialysate dataset except for the RSE% of covariate LBM on V_d_/F (RSE: 35.4%), the RSE% of other parameters was < 30%. CL/F decreased with the increase in Fe_baseline_ and V_d_/F increased with the increase of LBM. The RSE% for all the parameters in FPC pre-dialyzer administration data set was < 30%. The CL/F decreased with the increase in Fe_baseline_, whereas the V_d_/F increased with the increase in LBM. The influence of covariates on PK exposure parameters of all the final models (M1, M2, and M3) is presented in Online Resource 5, and the simulated drug concentration–time curve is shown in Fig. 1. When FPC was administered via dialysate (M2) and pre-dialyzer (M3) in patients with CKD-5HD (the target population) in Asians and non-Asians, the influence of each of the covariates on the PK exposures (AUC and C_max_) was < 50%. The effect of each of the covariates on PK exposures was analyzed separately, and the effect was not clinically significant. The effects of LBM and Fe_baseline_ on AUC and C_max_ were similar in Asian and non-Asian patient populations (Table 3). In Asians, the effects of LBM and Fe_baseline_ on PK exposures (AUC and C_max_) were 1 to 1.39 and 0.59 to 0.89, respectively, whereas the effects of LBM and Fe_baseline_ on AUC and C_max_ were 1 to 1.35 and 0.57 to 0.89, respectively, in non-Asian patients with CKD-5HD. In addition, the combination of covariates was analyzed and the effects of LBM combined with Fe_baseline_ on AUC and C_max_ on the PK exposure were 1.45 to 1.77. The GOF plots for each model showed that the model predictions were similar to the observations, especially for medium and high concentrations. For very low concentration, there was over-estimation to some extent in the model for healthy subjects. The high standard deviation (174 ng/mL) for M1 may also explain the discrepancy. However, owing to a relatively small low concentration sample points, which could result in the estimation bias, the predictions for PK exposures (C_max_ and AUC) can still be considered (Fig. 2). The regression trend line was close to the standard line, and the CWRES value was ± 6, which was evenly distributed on both sides of the coordinate axis [27]. Sensitivity analysis was performed to remove the outliers with the |CWRES| value > 3, and since there was no influence on all the model parameter estimations, the outliers were retained in the analysis data. For the bootstrapping evaluation, the proportion of successful minimizations were 98.8%, 97%, and 100% for M1, M2, and M3, respectively (Table 2). The median and 95% PI (2.5–97.5% percentile range) of bootstrap was similar to the parameter estimates and its 95% CI, indicating similar uncertainties in parameter estimates to a nonparametric bootstrap approach. The prediction-corrected VPC plots for all the models (Fig. 3) showed that observations were included within the range of concentrations simulated with the models.

**Table 2:** Parameter estimates for the final population PK model of FPC

**Parameters**	**Final Model**		**Bootstrap**	
	**Estimates (RSE%)**	**95% CI**	**Median**	**95% PI**
***FPC IV administration in healthy subjects***^a^			Bootstrap (988/1000)*	
**PK parameters**				
CL, L/h	0.477 (8.2)	0.400, 0.554	0.474	0.406, 0.555
V_d_, L	3.62 (7.0)	3.12, 4.12	3.60	3.16, 4.11
LBM on V_d_	3.26 (18.7)	2.06, 4.46	3.31	1.91, 4.48
Fe_.av_ on V_d_ (× 10^−4^)	7.71 (17.5)	5.06, 10.0	7.82	4.49, 9.77
θ_sex_ on V_d_	1.80 (30.1)	0.738, 2.86	1.81	0.970, 3.54
**Inter-individual variability**				
ω1 (CL, %)	42.8 (23)	0.235, 0.621	42.0	25.0, 60.7
ω2 (Vd, %)	33.8 (16.7)	0.227, 0.449	30.5	20.7, 41.5
**Residual error**				
σ (add), ng/ml	174 (8.9)	143, 204	173	144, 205
***FPC dialysate administration in patients with CKD-5HD***^b^			Bootstrap (970/1000)*	
**PK parameters**				
CL/F, L/h	0.982 (6.3)	0.861, 1.103	0.976	0.870, 1.12
V_d_/F, L	3.32 (3.5)	3.09, 3.55	3.33	3.11, 3.54
Fe_baseline_ on CL/F (× 10^−4^)	− 7.28(6.4)	− 8.19, − 6.37	− 7.07	− 9.62, − 3.19
LBM on V_d_/F	0.726 (35.4)	0.222, 1.23	0.712	0.172, 1.24
**Inter-individual variability**				
ω1 (CL, %)	41.5 (11.9)	31.8, 51.2	40.4	31.0 ~ 50.4
ω2 (V_d_, %)	18.1 (21.0)	10.7, 25.6	17.2	8.10, 24.0
**Residual error**				
σ1 (prop), %	23.6 (8.9)	0.195, 0.277	0.230	0.196, 0.279
σ2 (add), ng/ml	0.0220 (0.0)	-	0.0220	-
***FPC pre-dialyzer administration in patients with CKD-5HD***^c^			Bootstrap (1000/1000)*	
**PK parameters**				
CL/F, L/h	1.02 (5.9)	0.902, 1.14	1.02	0.924, 1.13
V_d_/F, L	3.57 (3.8)	3.30, 3.84	3.56	3.35, 3.80
Fe_baseline_ on CL/F (× 10^−4^)	− 7.02 (23.6)	− 10.2, − 3.77	− 6.88	− 9.90, − 4.11
LBM on V_d_/F	1.14 (28.2)	0.510, 1.77	1.11	0.598, 1.70
**Inter-individual variability**				
ω1 (CL, %)	36.6 (8.9)	30.2, 43.0	35.6	29.3, 42.0
ω2 (V_d_, %)	21.0 (23.2)	11.4, 30.5	19.9	7.99, 27.7
**Residual error**				
σ1 (prop), %	27.1 (7.8)	0.230, 0.312	0.271	0.237, 0.309
σ2 (add), ng/ml	0.0710 (0.0)	-	0.0710	-

*CI* confidence interval, *CKD-5HD* hemodialysis-dependent stage 5 chronic kidney disease, *CL* apparent clearance, *Fe*_*av*_ average serum total iron 6 h before the baseline period, *Fe*_*baseline*_ serum total iron at 0 h before administration, *IV* intravenous, *LBM* lean body mass, *PK* pharmacokinetic, *RSE* relative standard error, *V*_*d*_ apparent volume of distribution

^a^V_ind_ = V_TV_ × (1 + 0.000771 × [Fe_.av_ − 1110]) × (LBM/55.24)^3.26^ × COV_sex_

COV_male_ = 1; COV_female_ = 1 + θ_sex_ = 2.8

^b^CL_ind_ = CL_TV_ × (1 − 0.000728 × [Fe_baseline_ – 642.00]), where

V_ind_ = V_TV_ × (LBM/56.28)^0.726^

^c^CL_ind_ = CL_TV_ × (1 − 0.000702 × [Fe_baseline_ – 660.80])

Cl_ind_ is the individual predictive value of CL; CL_TV_ is a typical value of CL

V_ind_ = V_TV_ × (LBM/55.85)^1.14^

V_ind_ is the individual predictive value of V_d_; V_TV_ is a typical value of V_d_; CL_ind_ is the individual predictive value of CL and CL_TV_ is a typical value of CL

*The successful times in 1000 bootstrap minimizations

**Table 3:** Effect of covariates on PK parameters in Asians and non-Asians

**Covariates**	**C**_**max**_ **(ng/mL)**	**AUC**_**0-4 h or 0-3 h**_ **(h.ng/mL)**	**AUC**_**0-12 h**_ **(h.ng/mL)**	**AUC**_**0-24 h**_ **(h.ng/mL)**
*Population PK model of CKD-5HD patients treated with dialysate (Asians)*				
**LBM**				
5^th^ (39.01 kg)	1400.3	3413.22	7277.01	7548.96
95th (58.3 kg)	1201	2799.70	6923.82	7523.46
Ratio(5th/95th)	1.16	1.22	1.05	1.00
**Fe**_**baseline**_				
5^th^ (453.32 ng/mL)	1106.8	2723.91	5642.31	5817.10
95^th^ (1099.28 ng/mL)	1408.4	3205.99	8667.50	9820.80
Ratio (5th/95th)	0.78	0.85	0.65	0.59
**LBM + Fe**_**baseline**_				
5thLBM, 95th Fe_baseline_	1594.9	3727.58	9132.32	9886.06
95th LBM, 5th Fe_baseline_	1063	2577.76	5584.30	5814.94
Ratio	1.50	1.45	1.64	1.70
*Population PK model of pre dialyzer administration in CKD-5HD patients (Asians)*				
**LBM**				
5th (39.07 kg)	1596.9	2867.20	6773.90	6885.46
95th (58.23 kg)	1217.2	2058.06	6387.22	6862.41
Ratio (5th/95th)	1.31	1.39	1.06	1.00
**Fe**_**baseline**_				
5th (517.4 ng/mL)	1243.5	2186.61	5628.09	5786.78
95th (1146.6 ng/mL)	1488.6	2466.86	8548.65	9576.26
Ratio (5th/95th)	0.84	0.89	0.66	0.60
**LBM + Fe**_**baseline**_				
5th LBM, 95th Fe_baseline_	1842.7	3157.27	9159.71	9652.91
95th LBM, 5^th^ Fe_baseline_	1144	1974.28	5532.7	5782.38
Ratio	1.61	1.60	1.66	1.67
*Population PK model of CKD-5HD patients treated with dialysate (non-Asians)*				
**LBM**				
5th (45.73 kg)	1193.5	2950.71	6030.67	6201.30
95th (68.55 kg)	1031.5	2431.76	5780.49	6187.60
Ratio (5th/95th)	1.16	1.21	1.04	1.00
**Fe**_**baseline**_				
5th (280 ng/mL)	1003.9	2478.09	5087.95	5236.46
95th (1040 ng/mL)	1301.4	2953.52	8078.87	9212.54
Ratio(5th/95th)	0.77	0.84	0.63	0.57
**LBM + Fe**_**baseline**_				
5thLBM, 95th Febaseline	1452.8	3371.2	8476.34	9274.23
95thLBM, 5th Febaseline	951.12	2301.73	5018.32	5233.9
Ratio	1.53	1.46	1.69	1.77
*Population PK model of pre dialyzer administration in CKD-5HD patients (non-Asians)*				
**LBM**				
5th (48.4 kg)	1316.9	2325.27	5878.92	6027.96
95th (68.66 kg)	1026.9	1726.10	5527.55	6001.56
Ratio (5th/95th)	1.28	1.35	1.06	1.00
**Fe**_**baseline**_				
5th (317 ng/mL)	1091.2	1913.79	4980.84	5130.65
95th (1022 ng/mL)	1301	2153.28	7517.27	8448.65
Ratio (5th/95th)	0.84	0.89	0.66	0.61
**LBM + Fe**_**baseline**_				
5thLBM, 95th Fe_baseline_	1504.9	2543.26	7911.29	8506.17
95thLBM, 5th Fe_baseline_	972.35	1664.09	4854.89	5123.69
Ratio	1.55	1.53	1.63	1.66

*AUC*_*0-3 h*_ area under the plasma concentration–time curve from time 0 to 3 h in pre-dialyzer administration in CKD-5HD patients, *AUC*_*0-4 h*_ area under the plasma concentration–time curve from time 0 to 4 h inCKD-5HD patients treated with dialysate, *AUC*_*0-12 h*_ area under the plasma concentration–time curve from time 0 to 12 h, *AUC*_*0-24 h*_ area under the plasma concentration–time curve from time 0 to 24 h, CKD-5HD, hemodialysis-dependent stage 5 chronic kidney disease, *C*_*max*_ maximum observed plasma concentration, *Fe*_*baseline*_ serum total iron at 0 h before administration, *LBM* lean body mass, *PK* pharmacokinetic

### Influence of ethnicity on PK exposure parameters

The differences in the PK exposure between Asian and non-Asian populations after the adjustment for final model covariates are presented in Table 4. The geometric mean differences in the PK exposure parameters including maximum observed plasma concentration (C_max_) (ng/mL), area under the plasma concentration–time curve from time 0 to 4 h (AUC_0-4 h_) (h ng/mL), AUC from time 0 to 12 h (AUC_0-12 h_) (h ng/mL), AUC from time 0 to 24 h (AUC_0-24 h_) (h ng/mL), AUC from time 0 to infinity (AUC_0-∞_) (h ng/mL) in healthy subjects with FPC IV administration were < 40%, indicating that there was no significant ethnic difference. In patients with CKD-5HD who were administered FPC via dialysate or pre-dialyzer, the geometric mean difference in the PK exposure parameters in dialysate and pre-dialyzer blood circuit administration was < 20%, also indicating that there was no difference in ethnicity.

**Table 4:** Effect of ethnicity on PK parameters after covariate correction in the final population pharmacokinetic model

**Parameters**	**Ethnicity**	**Base model of PopPK**		**PopPK final model**	
		**GM LS mean**	**Asian-to-non-Asian** **Ratio (%)**	**GM LS Mean**	**Asian-to-non-Asian** **Ratio (%)**
**Intravenous administration in healthy subjects**					
C_max_ (ng/mL)	Asians	1738.4	184.2	1477.7	124.2
	Non-Asians	943.6		1189.9	
AUC_0-4 h_ (h ng/mL)	Asians	3903.6	196.8	3261.4	139.5
	Non-Asians	1983.5		2338.2	
AUC_0-12 h_ (h ng/mL)	Asians	10,981.7	163.5	9982.2	128.9
	Non-Asians	6716.3		7743.2	
AUC_0-24 h_ (h ng/mL)	Asians	12,729.5	114.8	12,322.1	100.9
	Non-Asians	11,087.5		12,210.2	
AUC_0-∞_ (h ng/mL)	Asians	12,929.9	97.6	12,869.9	97.6
	Non-Asians	13,241.3		13,189.8	
**Dialysate administration in patients with CKD-5HD**					
C_max_ (ng/mL)	Asians	1215.8	112.9	1116.8	101.5
	Non-Asians	1076.7		1100.8	
AUC_0-4 h_ (h.ng/mL)	Asians	2970.0	114.9	2742.9	104.3
	Non-Asians	2585.8		2630.0	
AUC_0-12 h_ (h.ng/mL)	Asians	6479.7	107.0	5787.0	93.3
	Non-Asians	6056.2		6202.0	
AUC_0-24 h_ (h.ng/mL)	Asians	6899.9	104.3	6074.9	90.1
	Non-Asians	6617.1		6741.6	
AUC_0-∞_ (h.ng/mL)	Asians	6955.4	103.9	6102.0	89.7
	Non-Asians	6693.9		6801.5	
**Pre-dialyzer administration in patients with CKD-5HD**					
C_max_ (ng/mL)	Asians	1390.9	121.5	1272.8	107.7
	Non-Asians	1145.1		1182.3	
AUC_0-3 h_ (h ng/mL)	Asians	2386.9	121.9	3290.1	108.0
	Non-Asians	1957.9		3046.2	
AUC_0-12 h_ (h ng/mL)	Asians	6638.9	116.3	6068.0	103.5
	Non-Asians	5706.3		5864.1	
AUC_0-24 h_ (h ng/mL)	Asians	7052.4	114.0	6390.8	101.6
	Non-Asians	6188.2		6289.6	
AUC_0-∞_ (h ng/mL)	Asians	7101.3	113.5	6411.0	101.2
	Non-Asians	6258.0		6336.6	

*AUC*_*0-4 h*_ area under the plasma concentration–time curve from time 0 to 4 h, *AUC*_*0-3 h*_ area under the plasma concentration–time curve from time 0 to 3 h, *AUC*_*0-12 h*_ area under the plasma concentration–time curve from time 0 to 12 h, *AUC*_*0-24 h*_ area under the plasma concentration–time curve from time 0 to 24 h, *AUC*_*0-∞*_ area under the plasma concentration–time curve from time 0 to infinity, *C*_*max*_ maximum observed plasma concentration, *CKD-5HD* hemodialysis-dependent stage 5 chronic kidney disease, *PopPK* population pharmacokinetic

## Discussion

The effectiveness of FPC in patients with CKD-5HD has been demonstrated in various studies, such as the physiological replenishment iron maintenance equivalency (PRIME) study, which showed that the administration of FPC via dialysate in patients with CKD-5HD maintained Hgb with reduced ESA dose requirements [18]. In addition, FPC delivered via dialysate during HD in the CRUISE 1 and 2 trials effectively replaced iron losses, maintained Hgb concentrations, did not increase iron stores, and exhibited a safety profile similar to that of placebo [19]. The PK exposure of FPC has been demonstrated in non-Asians [12, 20]. As the ethnicity influences the PK parameters of the drugs [28], in the present study, we compared the PK data obtained from healthy subjects as well as Asians and non-Asian patients with CKD-5HD to evaluate the potential influence of ethnicity on the exposure to FPC by population PK modeling analysis. Sampling points for patients were shorter, usually ≤ 12 h, while it was longer for healthy subjects, usually 24 h which may lead to large fluctuations in the low-concentration of blood drug concentration points, thereby resulting in higher IIV of base and final models in healthy subjects.

The results of the study revealed that the PK parameters were not significantly different between Asian and non-Asian populations. Furthermore, PK analyses by population PK model gave robust results, and no relevant influence of ethnicity on PK parameters was observed for both healthy subjects and patients with CKD-5HD. |CWRES| value of > 3 were considered as outliers and sensitivity analysis was performed to remove the outliers and there was no influence on all the model parameter estimations [27]. Overall, the PK parameters were comparable between Asian and non-Asian populations. Covariates such as Fe_baseline_ and LBM had slight impact on the PK exposure in both the Asian and non-Asian populations. The allometric exponents for the relationship to LBM in M2 and M3 were more consistent with an allometric expectation of an exponent of 1, but the allometric exponent in M1 was comparatively higher; this might be attributed to the small sample size; hence, validation in further studies with larger sample size is warranted to provide further insights. In the population PK model (M1) of healthy subjects who were given FPC IV, the influence of sex on model parameter V_d_ showed that the PK exposures of women were much higher than that of men. However, conclusion cannot be drawn on the impact of gender on PK parameters based on this result as the sample size of female subjects in the study was very small. Hence, the impact of gender on PK exposure requires substantiation in further studies with large sample size. The observed value of PK parameters in our study were in line with that of a phase I study which evaluated the PK and safety of IV FPC. The CL or CL/F in the current study were 0.474, 0.976, and 1.02 L/h in M1, M2, and M3, respectively. In healthy subjects with FPC, similar CL values were obtained that was in the range of 0.41 to 0.56 L/h [12].

In healthy subjects, PK exposures (C_max_ and AUC) decreased with increase in LBM and Fe_av_, while in both the patient populations, C_max_ and AUC decreased with increase in LBM and decrease in Fe_baseline_. Other factors such as gender, age, Fe_av_, and ethnicity had no influence on PK exposures in patients. The influence of LBM on PK exposures in patients was smaller than that in healthy subjects. The ratio of AUC_0-24_ for the 5th and 95th patient’s LBM was almost 1 indicating that dose regimen need not be adjusted as per patients’ weight. The Fe_baseline_ levels showed some influence both on Asian and non-Asian patients and a higher Fe_baseline_ levels may indicate a higher FPC exposures. Considering the influence of Fe_baseline_ was relatively small (a 5th/95th ratio of 0.57–0.89), the efficacy of FPC is unlikely to be influenced. Furthermore, there was no influence of LBM and Fe_baseline_ on PK exposures in both Asian and non-Asian patients (a 5^th^/95^th^ ratio of 1.45–1.70 and 1.46–1.77, respectively) even in the extreme covariate combinations for the highest or for the lowest exposures. In a clinical study by Pratt et al., the absorption of iron after the administration via dialysate roughly doubled with increasing age. In addition, iron exposure was greater after the administration via dialysate than after IV administration in patients with CKD-5HD [20]. However, in our study, a similar PK exposure was observed in administration via dialysate and pre-dialyzer administration in patients with CKD-5HD. Interestingly, in the IV administration model of healthy subjects, V_d_ of female subjects was 2.8 times higher than that of male subjects. This may be attributed to the fact that estrogens dilate and androgens constrict the renal microvasculature, where dilation and vasoconstriction increases and decreases the hematocrit levels, respectively [29].

According to the US trial, the 24-h fluctuation of serum total iron in healthy subjects during the baseline period is large, and there is no significant change in serum total iron in patients with CKD-5HD during the 24-h period. Therefore, it is reasonable for healthy subjects to use the corrected serum total iron in the baseline period, while for patients with CKD-5HD to directly use the serum total iron before each administration. In our study, the difference of serum total iron between Asian and non-Asian healthy subjects was significantly higher at 6 h before baseline, which reduced post 6 h with no significant difference between the 2 populations. We selected Fe._av_ as the final covariate to describe the baseline iron level, based on the fact that there was a strong collinearity between Fe._av_ and Fe._max_ (correlation coefficient: 0.975).

In this study, the PK of healthy subjects, patients with dialysate and pre-dialyzer administration were modelled separately by three models. In addition, a combined model with healthy subjects and patients together was explored, but the fitting for healthy subjects was poor and the added parameter estimation of F or between-trial variance could not solve the issue. Furthermore, only healthy subjects had i.v. data, so for a combined model, the bioavailability and the difference between healthy subjects and patients could not be distinguished. However, combining M2 and M3 data together is a good way to improve the power of covariates selection and support the result of M2 and M3 model. Hence, the modeling for patients’ data together was explored and showed similar results, including model parameters, inter-individual variability, and covariates influence as M2 and M3.

To our knowledge, this was the first population PK study to evaluate the effect of ethnicity on the PK of FPC in healthy subjects and patients with CKD-5HD. The results of our study revealed that ethnicity does not influence the PK parameters of FPC in both healthy subjects and patients with CKD-5HD. Further research should include the concentrations of various serum biomarkers of oxidative stress and inflammation as covariates into this PK model to determine their influence on ethnicity. A relatively small sample size for subject variability characterization poses as a limitation to the study. To provide further insights in to the generalizability of the results, studies with larger sample size is warranted. The effect of LBM and Fe_baseline_ on PK exposure in Asian and non-Asian populations were comparable; hence, data for either of these populations could be bridged to the other, thereby avoiding the duplication of effort and cost.

## Conclusion

In conclusion, we evaluated the ethnic differences in FPC PK profiles between Asian and non-Asian populations using a population PK model. No clinically relevant differences were found for the PK properties, indicating the clinically effective administration of FPC in CKD-5HD Asian population.

## Supplementary information

Below is the link to the electronic supplementary material.

Supplementary file1 (PDF 200 KB)

Supplementary file2 (PDF 176 KB)

Supplementary file3 (PDF 257 KB)

Supplementary file4 (PDF 205 KB)

Supplementary file5 (PDF 210 KB)

Supplementary file6 (TIF 25 KB)

## Data Availability

On request.
