# Supplementary material for: Ethnicity evaluation of ferric pyrophosphate citrate among Asian and Non-Asian populations: a population pharmacokinetics analysis
Source: Eur J Clin Pharmacol. 2022 Jun 17;78(9):1421–34. doi: 10.1007/s00228-022-03328-9 (PMC9365747; doi:10.1007/s00228-022-03328-9)
Supplement: Supplementary file 1 — Supplementary file1 (PDF 200 KB) [file 228_2022_3328_MOESM1_ESM.pdf]

Ethnicity Evaluation of Ferric Pyrophosphate Citrate among Asian and Non-Asian populations: A Population Pharmacokinetics Analysis

Linxiao Zhang[1*], Liangying Gan[2*], Kexin Li[3], Panpan Xie[3], Yan Tan[4], Gang Wei[4], Xiaojuan Yuan[5], Raymond Pratt [6], Yongchun Zhou[5], Ai-Min Hui[4], Yi Fang[2&], Li Zuo[2&], Qingshan Zheng[1&]

Affiliations

[1]Center for Drug Clinical Research, Shanghai University of Traditional Chinese Medicine, Shanghai, China

[2]Department of Nephrology, Peking University People's Hospital, Beijing, China

[3]Clinical trial center, Beijing hospital, National center of gerontology; Institute of geriatric medicine, Chinese academy of medical sciences, Assessment of Clinical Drugs Risk and Individual Application Key Laboratory, Beijing, China

[4]Global R&D Center, Shanghai Fosun Pharmaceutical Development, Co., Ltd, Shanghai, China

[5] Jiangsu Wanbang Biopharmaceuticals Co., Ltd., Xuzhou, China

[6] Rockwell Medical Inc. Wixom MI USA

[*]These authors contributed equally to this work.

[&]Corresponding author

Qingshan Zheng,

Center for Drug Clinical Research, Shanghai University of Traditional Chinese Medicine, Shanghai, China

E-mail: qingshan.zheng@drugchina.net

Li Zuo

Department of Nephrology, Peking University People's Hospital, Beijing, China

E-mail: ZuoLi@bjmu.edu.cn

Yi Fang

Department of Nephrology, Peking University People's Hospital, Beijing, China

E-mail: fygk7000@163.com

Supplementary Table 1. Studies included in the FPC population pharmacokinetic analysis set

Dosage Regimen	Sample Collection Time
CHN-FPC-14 healthy subjects (N = 14)	
Day 4: 6.5 mg of IV FPC was given for 4 hours	Day 3 (baseline period) (h)：0, 1, 2, 3, 4, 4.5, 5, 6, 8, 10, 12, 18<br>Day 4 (administration period) (h)：0, 1, 2, 3, 4, 4.5, 5, 6, 8, 10, 12, 18
CHN-FPC-21 patients with CKD-5HD patients (N = 12)	
Day 1: 95 μg/L of FPC was administered through dialysate for 4 hours<br>Day 3: 6.5 mg of FPC was given 3 hours before dialyzer	Day 1：0, 1, 2, 3, 3.5, 4, 4.5, 5, 6, 8, 10, 12<br>Day 3：0, 1, 2, 3, 3.5, 4, 4.5, 5, 6, 8, 10, 12
USA-FPC-12 healthy subjects (N = 12)	
Day 2: 6 mg of IV FPC was given for 4 hours<br>Day 3: 35 μg/kg of FPC, IV injection	Day 1 (baseline period) (h)：0, 1, 2, 3, 3.5, 4, 4.5, 5, 6, 8, 12, 16<br>Day 2 (administration period) (h)：0, 1, 2, 3, 3.5, 4, 4.5, 5, 6, 8, 12, 16<br>Day 3 (administration period) (h)：0, 3-5 min, 0.5, 1, 2, 3, 3.5, 4, 4.5, 5, 6, 8, 12, 16
USA-FPC-18 healthy subjects (N = 14)	
Days 2, 4, 6, 8 and 10: 6 mg of FPC orally<br>Day 12: 6.6 mg of IV FPC for 4 hours	Day 12 (administration period) (h)：0, 1, 2, 4, 6, 8, 12, 16, 24
USA-FPC-16 patients with CKD-5HD (N = 13)	
Day 3, 8 and 10: FPC administered randomly according to the following 3 dosing schedules:	Days 3, 8, and 10 (each administration period) (h)： 0, 1, 2, 3, 4, 5, 6, 8, 10, 12

1：2 μM (110 μg/L) of FPC, administered via dialysate for 4 hours<br>2：6.6 mg of FPC, 3 hours before dialyzer<br>3：6.6 mg of FPC, 3 hours after dialyzer	
USA-FPC-20 patients with CKD-5HD (N = 26)	
The second dialysis in the first week and the 2 dialysis in the second week were randomly administered according to the following 3 dosing schedules:<br>1：2 μM of FPC, administered via dialysate for 4 hours<br>2：6.5 mg of FPC, 3 hours before dialyzer<br>3：6.5 mg of FPC, 3 hours after dialyzer	Each administration period (h)： 0, 1, 2, 3, 3.5, 4, 4.5, 5, 6, 8, 10, 12

CKD-5HD, hemodialysis-dependent stage 5 chronic kidney disease.
