# Supplementary material for: Ethnicity evaluation of ferric pyrophosphate citrate among Asian and Non-Asian populations: a population pharmacokinetics analysis
Source: Eur J Clin Pharmacol. 2022 Jun 17;78(9):1421–34. doi: 10.1007/s00228-022-03328-9 (PMC9365747; doi:10.1007/s00228-022-03328-9)
Supplement: Supplementary file 2 — Supplementary file2 (PDF 176 KB) [file 228_2022_3328_MOESM2_ESM.pdf]

Ethnicity Evaluation of Ferric Pyrophosphate Citrate among Asian and Non-Asian populations: A Population Pharmacokinetics Analysis

Linxiao Zhang[1*], Liangying Gan[2*], Kexin Li[3], Panpan Xie[3], Yan Tan[4], Gang Wei[4], Xiaojuan Yuan[5], Raymond Pratt [6], Yongchun Zhou[5], Ai-Min Hui[4], Yi Fang[2&], Li Zuo[2&], Qingshan Zheng[1&]

Affiliations

[1]Center for Drug Clinical Research, Shanghai University of Traditional Chinese Medicine, Shanghai, China

[2]Department of Nephrology, Peking University People's Hospital, Beijing, China

[3]Clinical trial center, Beijing hospital, National center of gerontology; Institute of geriatric medicine, Chinese academy of medical sciences, Assessment of Clinical Drugs Risk and Individual Application Key Laboratory, Beijing, China

[4]Global R&D Center, Shanghai Fosun Pharmaceutical Development, Co., Ltd, Shanghai, China

[5] Jiangsu Wanbang Biopharmaceuticals Co., Ltd., Xuzhou, China

[6] Rockwell Medical Inc. Wixom MI USA

[*]These authors contributed equally to this work.

[&]Corresponding author

Qingshan Zheng,

Center for Drug Clinical Research, Shanghai University of Traditional Chinese Medicine, Shanghai, China

E-mail: qingshan.zheng@drugchina.net

Li Zuo

Department of Nephrology, Peking University People's Hospital, Beijing, China

E-mail: ZuoLi@bjmu.edu.cn

Yi Fang

Department of Nephrology, Peking University People's Hospital, Beijing, China

E-mail: fygk7000@163.com

Supplementary Table 2. Combined analysis study list

Study number	Dosing regimen	Reason for data consolidation
IV administration in healthy subjects		
CHN-FPC-14	FPC 6.5mg, IV for 4 h	1. Same route of administration<br>2. Same analysis method (serum total iron)<br>3. Same population (healthy subjects)<br>4. FPC has a short half-life (about 4.3 hours)<br>USA-FPC-18: Day 10 oral versus Day 12, there was no effect on the plasma concentration of the daily IV administration
USA-FPC-12	FPC 6 mg, IV for 4 h	
USA-FPC-18	FPC 6.6 mg, IV for 4 h	
Administered via dialysate in patients with CKD-5HD		
CHN-FPC-21	FPC 95 μg/L was administered via dialysate for 4 h	1. Same route of administration<br>2. Same analysis method (serum total iron)<br>3. Same population (patients with CKD-5HD)
USA-FPC-20	FPC 2 μM was administered via dialysate for 4 h	
USA-FPC-16	FPC 2 μM was administered via dialysate for 4 h	
Pre-dialyzer administration in patients with CKD-5HD		
CHN-FPC-21	FPC 6.5 mg, 3 h before dialyzer	1. Same route of administration<br>2. Same analysis method (serum total iron)<br>3. Same population (patients with CKD-5HD)
USA-FPC-20	FPC 6.5 mg, 3 h before dialyzer	
USA-FPC-16	FPC 6.6mg, 3 hours before dialyzer	

CKD-5HD, hemodialysis-dependent stage 5 chronic kidney disease; h, hours; IV, intravenous
