# Supplementary material for: Ethnicity evaluation of ferric pyrophosphate citrate among Asian and Non-Asian populations: a population pharmacokinetics analysis
Source: Eur J Clin Pharmacol. 2022 Jun 17;78(9):1421–34. doi: 10.1007/s00228-022-03328-9 (PMC9365747; doi:10.1007/s00228-022-03328-9)
Supplement: Supplementary file 3 — Supplementary file3 (PDF 257 KB) [file 228_2022_3328_MOESM3_ESM.pdf]

Ethnicity Evaluation of Ferric Pyrophosphate Citrate among Asian and Non-Asian populations: A Population Pharmacokinetics Analysis

Linxiao Zhang[1*], Liangying Gan[2*], Kexin Li[3], Panpan Xie[3], Yan Tan[4], Gang Wei[4], Xiaojuan Yuan[5], Raymond Pratt [6], Yongchun Zhou[5], Ai-Min Hui[4], Yi Fang[2&], Li Zuo[2&], Qingshan Zheng[1&]

Affiliations

[1]Center for Drug Clinical Research, Shanghai University of Traditional Chinese Medicine, Shanghai, China

[2]Department of Nephrology, Peking University People's Hospital, Beijing, China

[3]Clinical trial center, Beijing hospital, National center of gerontology; Institute of geriatric medicine, Chinese academy of medical sciences, Assessment of Clinical Drugs Risk and Individual Application Key Laboratory, Beijing, China

[4]Global R&D Center, Shanghai Fosun Pharmaceutical Development, Co., Ltd, Shanghai, China

[5] Jiangsu Wanbang Biopharmaceuticals Co., Ltd.，Xuzhou，China

[6] Rockwell Medical Inc. Wixom MI USA

[*]These authors contributed equally to this work.

[&]Corresponding author

Qingshan Zheng,

Center for Drug Clinical Research, Shanghai University of Traditional Chinese Medicine, Shanghai, China

E-mail: qingshan.zheng@drugchina.net

Li Zuo

Department of Nephrology, Peking University People's Hospital, Beijing, China

E-mail: ZuoLi@bjmu.edu.cn

Yi Fang

Department of Nephrology, Peking University People's Hospital, Beijing, China

E-mail: fygk7000@163.com

Supplementary Table 3. Covariate Exploration Process

	**Model description**	**OFV**	**Δ OFV**
**M1**			
Step 1: Forward selection (if △OFV< -3.84, covariates have a significant impact on parameters, P≤ 0.05)			
Base model (Model 3)		3964.82	
Model 5	Model 3 + Fe.av on V	3951.67	-13.15(compared to Model 3)
Step 2: Superimposed introduction (if △OFV< -3.84, covariates have a significant impact on parameters, P≤ 0.05)			
Model 6	Model 5 + RBC on CL	3941.12	-10.55 (compared to Model 5)
Model 7	Model 6 + Age on V	3932.69	-8.43 (compared to Model 6)
Model 8	Model 7 + LBM on V	3924.26	-8.43 (compared to Model 7)
Model 9	Model 8+ Sex on V	3918.12	-6.14 (compared to Model 8)
Step 3: Backward elimination (if △OFV> 6.63, covariates have a significant impact on parameters, P≤ 0.01)			
Model 10	Model 9 - Age on V	3920.583	2.461 (compared to Model 9)
Model 11	Model 9 - Sex on V	3924.259	6.137 (compared to Model 9)
Model 12	Model 9 - RBC on CL	3927.694	9.572 (compared to Model 9)

Model 13	Model 9 - LBM on V	3931.828	13.706 (compared to Model 9)
Model 14	Model 9 - Fe.av on V	3940.464	22.342 (compared to Model 9)
Model 15	Model 10 - Sex on V	3933.867	9.608 (compared to Model 10)
Model 16	Model 10 - RBC on CL	3930.317	6.058 (compared to Model 10)
Model 17	Model 10 - LBM on V	3941.133	16.874 (compared to Model 10)
Model 18	Model 10 - Fe.av on V	3943.372	19.113 (compared to Model 10)
Model 19	Model 16 - Sex on V	3944.146	13.829 (compared to Model 16)
Model 20	Model 16 - LBM on V	3951.641	21.324 (compared to Model 16)
Model 21	Model 16 - Fe.av on V	3953.42	23.103 (compared to Model 16)
**M2**			
Step 1: Forward selection (if △OFV< -3.84, covariates have a significant impact on parameters, P≤ 0.05)			
Base Model (Model 3)		5026.200	
Model 4	Model 3 + LBM on CL	5026.200	-0.000
Model 5	Model 3 + LBM on V	5017.382	-8.818
Step 2: Superimposed introduction (if △OFV< -3.84, covariates have a significant impact on parameters, P≤ 0.05)			
Model 6	Model 5 + $Fe_{Baseline}$ on CL	5008.222	-9.160 (compared to Model 5)
Model 7	Model 6 + ALT on CL	5001.684	-6.538 (compared to Model 6)
Model 8	Model 7 + TC on CL	4996.398	-5.285 (compared to Model 7)
Step 3: Backward elimination (if △OFV> 6.63, covariates have a significant impact on parameters, P≤ 0.01)			
Model 9	Model 8 – ALT on CL	5004.404	+8.006 (compared to Model 8)
Model 10	Model 8 –$Fe_{Baseline}$ on CL	5006.470	+10.071 (compared to Model 8)
Model 11	Model 8 – LBM on V	5005.071	+8.673 (compared to Model 8)
Model 12	Model 8 – TC on CL	5001.684	+5.285 (compared to Model 8)
Model 13	Model 12 –$Fe_{Baseline}$ on CL	5012.276	+10.592 (compared to Model 12)
Model 14	Model 12 – ALT on CL	5008.222	+6.538 (compared to Model 12)
Model 15	Model 12– LBM on V	5010.361	+8.677 (compared to Model 12)
Model 16	Model 14 –$Fe_{Baseline}$ on CL	5017.382	+9.160 (compared to Model 14)
Model 17	Model 14 – LBM on V	5016.911	+8.689 (compared to Model 14)
**M3**			
Step 1: Forward selection (if △OFV< -3.84, covariates have a significant impact on parameters, P≤ 0.05)			
Base Model (Model 3)		5278.560	
Model 4	Model 3 + LBM on CL	5263.300	-15.260

Step 2: Superimposed introduction (if △OFV< -3.84, covariates have a significant impact on parameters, P≤ 0.05)			
Model 5	Model 4 + $Fe_{Baseline}$ on CL	5252.512	-10.788 (compared to Model 4)
Model 6	Model 5 + TC on V	5246.018	-6.493 (compared to Model 5)
Step 3: Backward elimination (if △OFV> 6.63, covariates have a significant impact on parameters, P≤ 0.01)			
Model 7	Model 6 – LBM on CL	5264.991	+18.973 (compared to Model 6)
Model 8	Model 6 –$Fe_{Baseline}$ on V	5257.247	+11.229 (compared to Model 6)
Model 9	Model 6 – TC on V	5252.512	+6.493 (compared to Model 6)
Model 10	Model 9 – LBM on CL	5267.091	+14.579 (compared to Model 9)
Model 11	Model 9 –$Fe_{Baseline}$ on V	5263.300	+10.788 (compared to Model 9)
