# Supplementary material for: Ethnicity evaluation of ferric pyrophosphate citrate among Asian and Non-Asian populations: a population pharmacokinetics analysis
Source: Eur J Clin Pharmacol. 2022 Jun 17;78(9):1421–34. doi: 10.1007/s00228-022-03328-9 (PMC9365747; doi:10.1007/s00228-022-03328-9)
Supplement: Supplementary file 4 — Supplementary file4 (PDF 205 KB) [file 228_2022_3328_MOESM4_ESM.pdf]

Ethnicity Evaluation of Ferric Pyrophosphate Citrate among Asian and Non-Asian populations: A Population Pharmacokinetics Analysis

Linxiao Zhang[1*], Liangying Gan[2*], Kexin Li[3], Panpan Xie[3], Yan Tan[4], Gang Wei[4]，Xiaojuan Yuan[5]，Raymond Pratt [6]，Yongchun Zhou[5], Ai-Min Hui[4]，Yi Fang[2&], Li Zuo[2&], Qingshan Zheng[1&]

Affiliations

[1]Center for Drug Clinical Research, Shanghai University of Traditional Chinese Medicine, Shanghai, China

[2]Department of Nephrology, Peking University People's Hospital, Beijing, China

[3]Clinical trial center, Beijing hospital, National center of gerontology; Institute of geriatric medicine, Chinese academy of medical sciences, Assessment of Clinical Drugs Risk and Individual Application Key Laboratory, Beijing, China

[4]Global R&D Center, Shanghai Fosun Pharmaceutical Development, Co., Ltd, Shanghai, China

[5] Jiangsu Wanbang Biopharmaceuticals Co., Ltd.，Xuzhou，China

[6] Rockwell Medical Inc. Wixom MI USA

[*]These authors contributed equally to this work.

[&]Corresponding author

Qingshan Zheng,

Center for Drug Clinical Research, Shanghai University of Traditional Chinese Medicine, Shanghai, China

E-mail: qingshan.zheng@drugchina.net

Li Zuo

Department of Nephrology, Peking University People's Hospital, Beijing, China

E-mail: ZuoLi@bjmu.edu.cn

Yi Fang
Department of Nephrology, Peking University People's Hospital, Beijing, China
E-mail: fygk7000@163.com

Supplementary Table 4. Baseline demographic characteristics of M1, M2 and M3 models.

	M1			M2			M3		
	CHN-FPC-14 (N = 14)	USA-FPC-12 (N = 12)	USA-FPC-18 (N = 14)	CHN-FPC-21 (N = 12)	USA-FPC-16 (N = 13)	USA-FPC-20 (N = 25)	CHN-FPC-21 (N = 12)	USA-FPC-16 (N = 13)	USA-FPC-20 (N = 26)
Gender									
Male/Female	13/1	10/2	12/2	9/3	11/2	20/5	9/3	11/2	21/5
Age(yr)									
Mean±SD	30.8±5.92	38.4±8.99	39.1±11.99	54.3±16.47	49.2±8.84	53.9±8.26	54.3±16.47	49.2±8.84	53.7±8.17
Min, max	19, 40	24, 54	21, 62	25, 77	34, 63	36, 68	25, 77	34, 63	36, 68
Weight(kg)									
Mean±SD	68.12±6.942	84.48±12.892	73.71±12.937	69.38±10.243	97.79±19.979	84.92±14.417	69.28±10.207	98.01±19.890	84.28±14.262
Min, max	57.3, 76.6	65, 104.9	53.3, 95.3	56.7, 91.1	65, 129	51.3, 122.8	57, 91.1	64.3, 128.4	53, 123.1
RBC (x10^{12}/L)									
Mean±SD	5.082±0.3311	4.898±0.4289	4.799±0.3744	3.710±0.4852	4.091±0.5683	3.936±0.5108	3.710±0.4852	4.091±0.5683	3.931±0.5013
Min, max	4.42, 5.56	4.02, 5.46	4.22, 5.5	3.12, 4.57	3.31, 5.44	3.07, 4.87	3.12, 4.57	3.31, 5.44	3.07, 4.87
HB (g/dL)									
Mean±SD	15.71±1.000	14.54±1.251	14.63±0.856	11.90±1.271	12.31±1.211	11.71±1.505	11.90±1.271	12.31±1.211	11.70±1.476
Min, max	13.3, 18	12.9, 16.6	13.2, 16.1	9.9, 14.4	10.5, 15.2	9.2, 14.3	9.9, 14.4	10.5, 15.2	9.2, 14.3
PLT (x10^{9}/L)									
Mean±SD	267.9±54.26	220.5±52.03	226.9±36.38	188.3±50.36	154.8±46.34	190.0±67.13	188.3±50.36	154.8±46.34	190.2±65.77
Min, max	167, 366	148, 333	152, 272	107, 244	87, 252	94, 339	107, 244	87, 252	94, 339
CrCL(ml/min)									
Mean±SD	137.764±20.7932	114.537±22.8638	103.741±21.8523	6.958±0.9974	14.444±6.3883	12.538±4.4446	6.948±1.0002	14.489±6.4753	12.618±4.3340
Min, max	98.73, 166.06	93.12, 176.56	64.96, 162.15	5.52, 8.93	6.9, 27.6	6.65, 26.55	5.56, 8.93	6.93, 28.08	6.87, 26.04
AST (U/L)									
Mean±SD	19.1±4.07	19.2±3.19	21.1±4.57	14.2±3.51	21.2±12.23	18.2±5.10	14.2±3.51	21.2±12.23	18.2±5.00
Min, max	13, 27	14, 23	15, 29	10, 23	11, 54	10, 33	10, 23	11, 54	10, 33
ALT (U/L)									
Mean±SD	19.9±10.07	21.8±6.21	21.6±8.11	13.6±7.28	22.9±19.87	13.0±6.85	13.6±7.28	22.9±19.87	13.2±6.75
Min, max	8, 44	13, 30	10, 41	4, 28	10, 84	3, 33	4, 28	10, 84	3, 33
ALP (U/L)									
Mean±SD	84.6±16.23	63.3±15.14	66.9±19.50	91.9±29.46	121.9±66.72	96.5±40.70	91.9±29.46	121.9±66.72	95.8±40.06
Min, max	66, 115	42, 87	39, 119	50, 153	42, 313	34, 186	50, 153	42, 313	34, 186
TB (mg/dL)									
Mean±SD	0.648±0.2218	0.442±0.2065	0.650±0.2139	0.446±0.1648	0.454±0.2504	0.344±0.1417	0.446±0.1648	0.454±0.2504	0.346±0.1392
Min, max	0.4, 1.09	0.2, 0.9	0.4, 1.2	0.27, 0.76	0.1, 1.1	0.1, 0.7	0.27, 0.76	0.1, 1.1	0.1, 0.7
TC (mg/dL)									

	M1			M2			M3		
	CHN-FPC-14 (N = 14)	USA-FPC-12 (N = 12)	USA-FPC-18 (N = 14)	CHN-FPC-21 (N = 12)	USA-FPC-16 (N = 13)	USA-FPC-20 (N = 25)	CHN-FPC-21 (N = 12)	USA-FPC-16 (N = 13)	USA-FPC-20 (N = 26)
Mean±SD	164.375±21.2094	178.583±25.4789	188.143±40.7353	155.903±27.8882	155.231±34.6269	151.600±33.3841	155.903±27.8882	155.231±34.6269	150.808±32.9582
Min, max	123.74, 210.75	140, 216	125, 264	88.55, 185.23	114, 232	88, 210	88.55, 185.23	114, 232	88, 210
CRP (mg/dL)									
Mean±SD	NA±NA	1.16±0.403	1.49±0.773	0.4494±0.4075	0.8131±0.8136	0.8580±0.6627	0.4494±0.4075	0.8131±0.8136	0.9188±0.7196
Min, max	NA, NA	1, 2.4	1, 3.1	0.079, 1.429	0.03, 2.2	0.01, 2.35	0.079, 1.429	0.03, 2.2	0.01, 2.44
$Fe_{.av}$ (ng/mL)									
Mean±SD	946.40±221.420	1170.76±352.790	1425.59±554.665	-	-	-	-	-	-
Min, max	583.1, 1250.2	490.7, 1732.9	693.3, 2828.6	-	-	-	-	-	-
$Fe_{.cmax}$(ng/mL)									
Mean±SD	1054.40±217.147	1367.92±402.200	1750.86±648.124	-	-	-	-	-	-
Min, max	694.4, 1360.8	584, 1949	1074, 3301	-	-	-	-	-	-
Baseline Fe_{tot}(ng/mL)									
Mean±SD	-	-	-	798.93±226.913	563.85±194.231	613.20±246.183	794.73±222.998	594.62±206.180	656.15±246.480
Min, max	--	-	-	425.6, 1204	280, 860	210, 1100	369.6, 1254.4	320, 920	230, 1320
LBM (kg)									
Mean±SD	51.8126±5.0107	59.1027±8.7638	54.0152±6.8216	50.5911±6.6230	62.2921±8.6279	57.2599±5.9163	50.5541±6.5864	62.3638±8.4974	57.1084±5.6786
Min, max	37.919, 56.3103	41.7089, 70.3564	37.6236, 64.2388	37.70385, 61.13725	48.99525, 76.98874	43.15171, 67.71113	37.80009, 61.13725	48.71417, 76.76007	43.8338, 67.74489

ALP, alkaline phosphatase; ALT, alanine transaminase; AST, aspartate aminotransferase; CrCL, creatinine clearance; AST, CRP, c-reactive protein; $Fe_{.av}$, average serum total iron at 6 hours before the baseline period; Fe_{to}, total iron; HB, hemoglobin; FPC, ferric pyrophosphate citrate; LBM, lean body mass; M1, healthy subjects; M2: CKD-5HD patients treated with FPC dialysate; M3: CKD-5HD patients treated with pre-dialyzer FPC; PLT, platelets; RBC, red blood cells; SD, standard deviation; TB, total bilirubin; TC, total cholesterol
