# Supplementary material for: Ethnicity evaluation of ferric pyrophosphate citrate among Asian and Non-Asian populations: a population pharmacokinetics analysis
Source: Eur J Clin Pharmacol. 2022 Jun 17;78(9):1421–34. doi: 10.1007/s00228-022-03328-9 (PMC9365747; doi:10.1007/s00228-022-03328-9)
Supplement: Supplementary file 5 — Supplementary file5 (PDF 210 KB) [file 228_2022_3328_MOESM5_ESM.pdf]

Ethnicity Evaluation of Ferric Pyrophosphate Citrate among Asian and Non-Asian populations: A Population Pharmacokinetics Analysis

Linxiao Zhang[1*], Liangying Gan[2*], Kexin Li[3], Panpan Xie[3], Yan Tan[4], Gang Wei[4], Xiaojuan Yuan[5], Raymond Pratt [6], Yongchun Zhou[5], Ai-Min Hui[4], Yi Fang[2&], Li Zuo[2&], Qingshan Zheng[1&]

Affiliations

[1]Center for Drug Clinical Research, Shanghai University of Traditional Chinese Medicine, Shanghai, China

[2]Department of Nephrology, Peking University People's Hospital, Beijing, China

[3]Clinical trial center, Beijing hospital, National center of gerontology; Institute of geriatric medicine, Chinese academy of medical sciences, Assessment of Clinical Drugs Risk and Individual Application Key Laboratory, Beijing, China

[4]Global R&D Center, Shanghai Fosun Pharmaceutical Development, Co., Ltd, Shanghai, China

[5] Jiangsu Wanbang Biopharmaceuticals Co., Ltd., Xuzhou, China

[6] Rockwell Medical Inc. Wixom MI USA

[*]These authors contributed equally to this work.

[&]Corresponding author

Qingshan Zheng,

Center for Drug Clinical Research, Shanghai University of Traditional Chinese Medicine, Shanghai, China

E-mail: qingshan.zheng@drugchina.net

Li Zuo

Department of Nephrology, Peking University People's Hospital, Beijing, China

E-mail: ZuoLi@bjmu.edu.cn

Yi Fang

Department of Nephrology, Peking University People's Hospital, Beijing, China

E-mail: fygk7000@163.com

Supplementary Table 5. Effects of covariates on PK exposure parameters

Covariate	C_{max} (ng/mL)	$AUC_{0\text{-}4h}$ (h·ng/mL)	$AUC_{0\text{-}12h}$ (h·ng/mL)	$AUC_{0\text{-}24h}$ (h·ng/mL)
FPC IV administration in healthy subjects				
**LBM**				
5th (41.5 kg)	2513.8	6116.8	13110.9	13617.5
95th (65.9 kg)	874.1	1834.5	7109.8	10949.4
Ratio (5th/95th)	2.8	3.3	1.8	1.2
**Fe_{av}**				
5th (613.7 ng/mL)	991.7	2097.0	7833.0	11563.0
95th (1976.9 ng/mL)	407.2	831.7	3708.1	6857.0
Ratio (5th/95th)	2.4	2.5	2.1	1.6
**Sex**				
Male	1395.6	3035.2	9935.7	12867.5
Female	584.5	1205.7	5102.5	8780.6
Ratio (male/female)	2.3	2.5	1.9	1.4
Dialysate administration in patients with CKD-5HD				
**LBM**				
5th (44.31 kg)	1250.0	3068.5	6408.3	6619.3
95th (68.17 kg)	1060.0	2485.4	6092.5	6598.1

Ratio ($5^{th}/95^{th}$)	1.1	1.2	1.0	1
**$Fe_{baseline}$**				
5th (324.5 ng/mL)	1030.9	2545.2	5222.4	5374.2
95th (1040 ng/mL)	1324.4	3015.2	8145.1	9224.8
Ratio ($5^{th}/95^{th}$)	0.7	0.8	0.6	0.5
Pre-dialyzer administration in patients with CKD-5HD				
**LBM**				
5th (44.7 kg)	1419.1	2518.8	6232.0	6370.4
95th (67.83 kg)	1054.7	1765.2	5785.3	6334.6
Ratio ($5^{th}/95^{th}$)	1.3	1.4	1.0	1.0
**$Fe_{baseline}$**				
5th (335 ng/mL)	1124.7	1982.3	5050.0	5184.0
95th (1094.2 ng/mL)	1370.7	2263.5	8006.6	9052.8
Ratio ($5^{th}/95^{th}$)	0.8	0.8	0.6	0.5

$AUC_{0\text{-}4h}$, area under the plasma concentration–time curve from time 0 to 4 hours; $AUC_{0\text{-}12h}$, area under the plasma concentration–time curve from time 0 to 12 hours; $AUC_{0\text{-}24h}$, area under the plasma concentration–time curve from time 0 to 24 hours; C_{max}, maximum observed plasma concentration; Fe_{av}, average serum total iron 6 hours before the baseline period; $Fe_{baseline}$, serum total iron at 0 hours before administration; IV, intravenous; LBM, lean body mass; PK, pharmacokinetic
