# Supplementary figures and images for: Ethnicity evaluation of ferric pyrophosphate citrate among Asian and Non-Asian populations: a population pharmacokinetics analysis
Source: Eur J Clin Pharmacol. 2022 Jun 17;78(9):1421–34. doi: 10.1007/s00228-022-03328-9 (PMC9365747; doi:10.1007/s00228-022-03328-9)

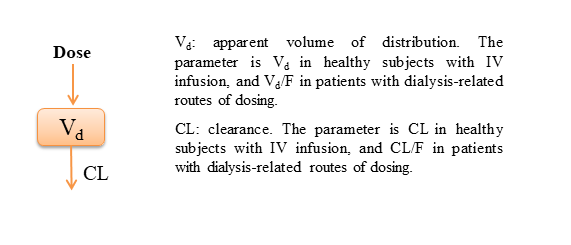

Supplement: Supplementary file 6 — Supplementary file6 (TIF 25 KB) [file 228_2022_3328_MOESM6_ESM.tif]
